# Application of loop analysis for evaluation of malaria control interventions

**DOI:** 10.1186/1475-2875-13-140

**Published:** 2014-04-09

**Authors:** Junko Yasuoka, Masamine Jimba, Richard Levins

**Affiliations:** 1 Department of Community and Global Health, The University of Tokyo, 7-3-1 Hongo, Bunkyo-ku, Tokyo 113-0033, Japan; 2 Department of Global Health and Population, Harvard School of Public Health, 665 Huntington Avenue, Boston, MA 02115, USA

**Keywords:** Qualitative analysis, Malaria control, Community-based intervention

## Abstract

**Background:**

Despite continuous efforts and recent rapid expansion in the financing and implementation of malaria control interventions, malaria still remains one of the most devastating global health issues. Even in countries that have been successful in reducing the incidence of malaria, malaria control is becoming more challenging because of the changing epidemiology of malaria and waning community participation in control interventions. In order to improve the effectiveness of interventions and to promote community understanding of the necessity of continued control efforts, there is an urgent need to develop new methodologies that examine the mechanisms by which community-based malaria interventions could reduce local malaria incidence.

**Methods:**

This study demonstrated how the impact of community-based malaria control interventions on malaria incidence can be examined in complex systems by qualitative analysis combined with an extensive review of literature. First, sign digraphs were developed through loop analysis to analyse seven interventions: source reduction, insecticide/larvicide use, biological control, treatment with anti-malarials, insecticide-treated mosquito net/long-lasting insecticidal net, non-chemical personal protection measures, and educational intervention. Then, for each intervention, the sign digraphs and literature review were combined to analyse a variety of pathways through which the intervention can influence local malaria incidence as well as interactions between variables involved in the system. Through loop analysis it is possible to see whether increases in one variable qualitatively increases or decreases other variables or leaves them unchanged and the net effect of multiple, interacting variables.

**Results:**

Qualitative analysis, specifically loop analysis, can be a useful tool to examine the impact of community-based malaria control interventions. Without relying on numerical data, the analysis was able to describe pathways through which each intervention could influence malaria incidence on the basis of the qualitative patterns of the interactions between variables in complex systems. This methodology is generalizable to various disease control interventions at different levels, and can be utilized by a variety of stakeholders such as researchers, community leaders and policy makers to better plan and evaluate their community-based disease control interventions.

## Background

Malaria remains one of the most devastating global health threats. In spite of continuous efforts and recent rapid expansion in the financing and implementation of malaria control programmes, millions of people still suffer from a lack of access to preventive measures, diagnostic testing and quality-assured treatment [1]. The Global Malaria Eradication Campaign in the 1950s and 60s failed due to its assumption that malaria eradication could be achieved by a one-size-fits-all strategy rather than tailor-made interventions that take local contexts into account [2]. Yet current malaria control relies heavily on such a failed strategy, using a limited number of tools, particularly anti-malarial drugs and insecticide-treated mosquito nets/long-lasting insecticidal nets (ITN/LLIN), both of which have become less effective because of resistance [1]. Vector control follows a similar strategy and has not been successful either mainly because of continued heavy dependence on chemical spraying, which has led to vectors’ resistance to insecticides [3,4]. Furthermore, the lack of intersectoral cooperation, interdisciplinary approaches and community participation has been impeding sustainability in malaria control efforts [5-7]. Consequently, these challenges have led to a growing interest in formulating new approaches for developing, delivering and maintaining malaria control, especially in areas with high and/or unstable transmission [3,8,9].

When developing new strategies, possible consequences of each intervention need to be examined within a complex system. A majority of current efforts to plan and conduct interventions and to evaluate their effectiveness are narrowly focused on direct associations between a limited number of factors. For example, there has been a heavy reliance on insecticide to control malaria vectors mainly due to the belief in the single, direct aspect of the insecticide’s impact (lethal effect) on the pest. However, it does not necessarily work that way for at least three reasons: 1) a decrease in the vector population can decrease the predator population by affecting the availability of food for the predators; 2) insecticide directly reduces the predators of the vector; and, 3) natural selection in the vector population rapidly builds up resistance to the insecticide [10]. Therefore, there is a need for a methodology that enables us to better understand complex systems, to examine the associations and correlations among a variety of factors involved, and to foresee how unexpected consequences might occur.

Furthermore, the methodology needs to be utilized not only by researchers, policy makers and programme and project implementers, but also by community members. Community participation is a key to the success of malaria control interventions at the community level, and obtaining support and enthusiasm for participation is expected to become more challenging as malaria transmission becomes lower [2,11,12]. However, the lack of perceived risk of disease and inadequate knowledge about the reasons for conducting interventions are the two most influential factors negatively affecting acceptability of communicable disease control and elimination programmes [2]. Therefore, it is vital to promote community understanding of the whole picture of malaria control interventions conducted in the community, especially about the role that community members play in the full system and how their actions and efforts might lead to a decrease in local malaria incidence. In this study, sign digraphs were developed using a method of qualitative modelling, loop analysis, to demonstrate how community-based malaria control could work in complex systems.

## Methods

### Loop analysis to develop sign digraphs

Details of the procedure to conduct loop analysis have been described elsewhere [10,13-15], and the method has been utilized as a standard approach mainly in ecology and biology [16-20]. Briefly, loop analysis consists of the analysis of sign digraphs, which show whether increases in one variable qualitatively increases or decreases other variables, or leaves them unchanged. It does not require precise quantitative interaction rates for the system being studied. The directions of associations between variables can be shown as a community matrix, which is a set of signs of interaction rates for each pair of variables [21]. The following matrix is an example of two variables, x_1_ and x_2_. The effect from a variable x_2_ on x_1_ (a_12_) is negative (−), whereas that of variable x_1_ on x_2_ (a_21_) is positive (+). The variable x_2_ also has a negative effect on itself when excessive levels are reached, which is called self-damping (a_22_). Here, positive or negative entries (+1 or −1) do not mean that the magnitude of the interactions between variables is equal.

The above matrix corresponds to a sign digraph, using symbols of loop analysis, as shown below.

**Figure 1:**
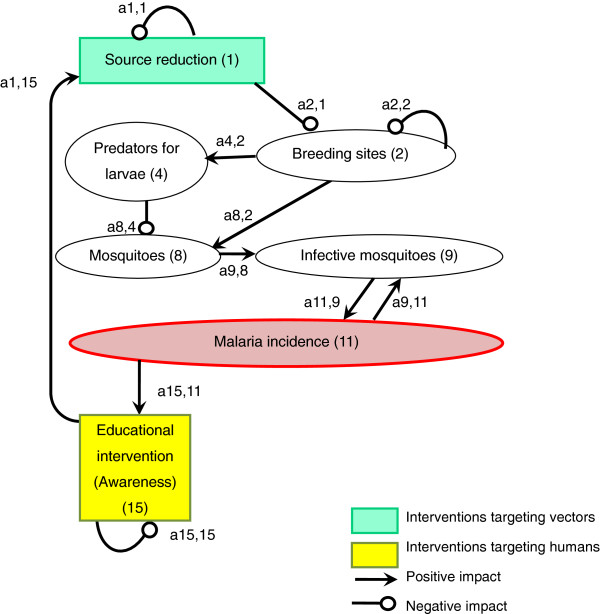
Source reduction.

**Figure 2:**
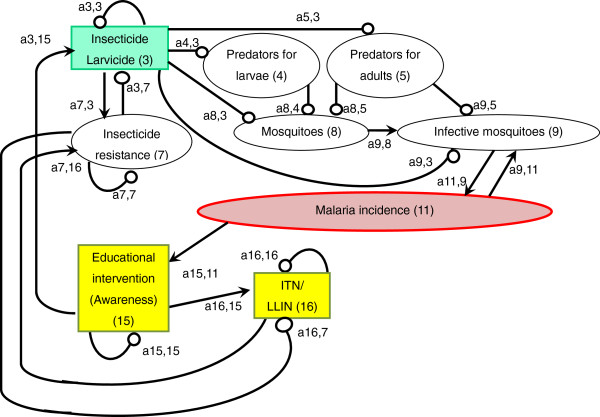
Insecticide and larvicide use.

**Figure 3:**
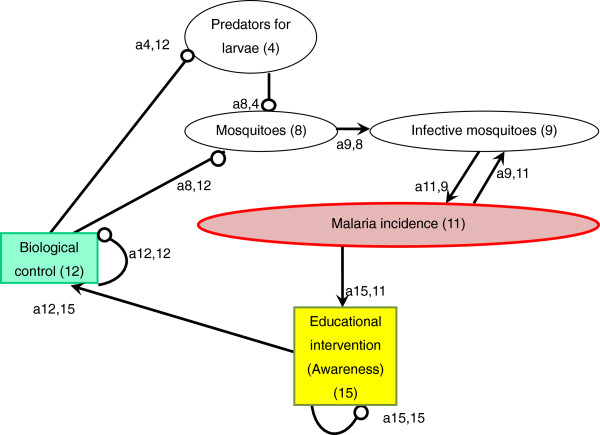
Biological control.

**Figure 4:**
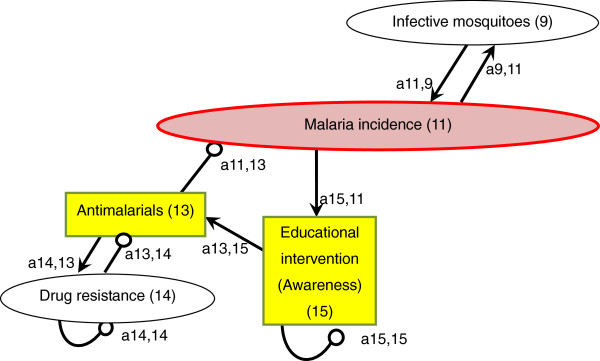
Anti-malarials.

**Figure 5:**
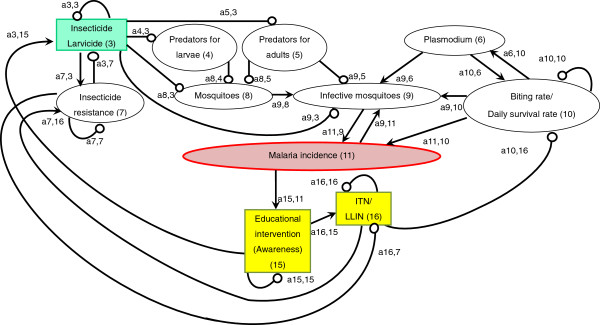
Insecticide-treated mosquito nets/Long-lasting insecticidal nets.

**Figure 6:**
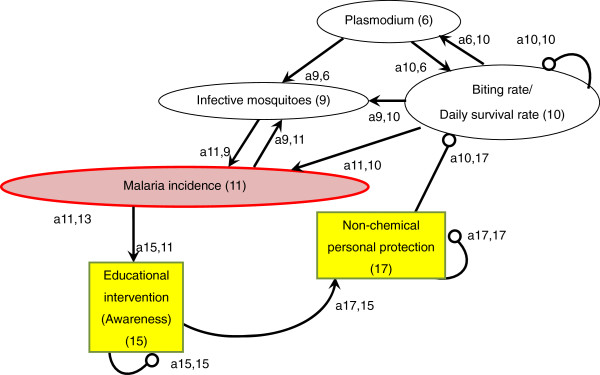
Non-chemical personal protection measures.

**Figure 7:**
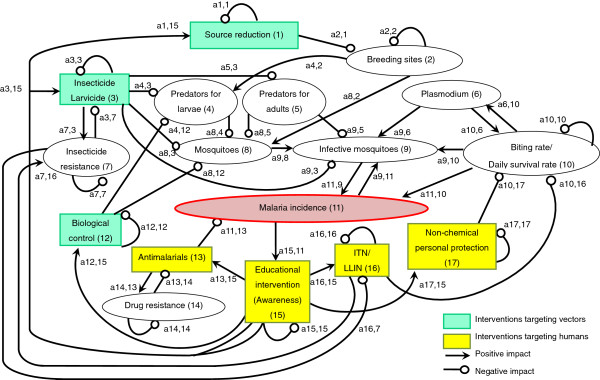
Educational intervention (awareness).

An arrow from one variable to another denotes that the variable has a positive effect on the other, while a line ending in a circle denotes a negative effect on the other variable or itself. Here, the arrow a_21_ from x_1_ to x_2_ indicates a positive effect, and the line ending in a circle (a_12_) from x_2_ to x_1_ indicates a negative effect. Variable x_2_ is self-damped, represented by a line ending in a circle at itself (a_22_). (In Figures 1, 2, 3, 4, 5, 6 and 7, a comma is inserted between two numbers of variables for clarity.)

### Steps taken to develop sign digraphs for this study

First, out of the interventions described in the World Malaria Report 2012 [1], seven major interventions, which are currently conducted at community level and involve community residents and/or community health workers, were selected. They include source reduction, insecticide/larvicide use (including indoor residual spraying (IRS), biological control, treatment with anti-malarials, ITN/LLIN, non-chemical personal protection measures, and educational intervention. Pathways through which each intervention could change local malaria incidence were identified. A sign digraph was then created for each intervention with the loop analysis method, connecting variables involved in the pathways. If any of the other six interventions were relevant to the intervention, they were included in the same sign digraph. (For example, all sign digraphs include the variable “educational intervention” because it has a positive impact on other interventions.) Each variable was randomly numbered so each pathway can be described in the text simply using the numbers. (For example, a negative impact of a variable “Source reduction (1)” on another variable “Breeding sites (2)” is described as “a_21_(−)” in text). The decision on which sign to be used (positive, negative, or null) for each connection between variables was made based on a literature review of each intervention. Developing sign digraphs by combining the method of loop analysis and literature review is a unique approach taken in this study to qualitatively assess the mechanisms of how community-based malaria control intervention could influence local malaria incidence. Unlike other approaches to map out interventions, such as the intervention mapping approach [22], loop analysis examines the interactions between variables involved in the interventions of interest.

### Literature search

Peer-reviewed scientific literature on community-based malaria control interventions was searched through Pubmed/MEDLINE using keywords “malaria” and “community” as major subjects. To search the literature for each intervention, these keywords were combined with terms referring to different types of interventions and related variables, including “source reduction”, “insecticide”, “insecticide residual spraying”, “larvicide”, “biological control”, “mosquito fish”, “anti-malarials”, “education”, “awareness”, “bed net”, “insecticide-treated net”, “personal protection”, “house”, and “resistance”. Reference lists in the literature found were reviewed for additional articles. Then, relevant articles that explain the associations between variables in the sign digraphs were selected. Although information or data from primary sources were preferred, more general reviews and texts were also consulted.

## Results

### Source reduction

Source reduction provides the dual benefits of reducing the number of both indoor- and outdoor-biting mosquitoes [23] (Figure 1). In the sign digraph, the impact of source reduction on infective mosquitoes is shown by three lines: first, a negative line from “Source reduction (1)” to “Breeding sites (2)”; second, a positive arrow from “Breeding sites (2)” to “Mosquitoes (8)”; and third, a positive arrow from “Mosquitoes (8)” to “Infective mosquitoes (9)”. All over, the direction of the impact of source reduction on infective mosquitoes can be “negative times positive times positive = negative” [a2,1(−) x a8,2(+) x a9,8(+) = infective mosquitoes(−)] [24]. Source reduction activities include elimination of water-holding containers, filling standing water where mosquitoes oviposit and develop, field drainage, and cleaning and flushing of irrigation canals [5,25-27]. These activities can prevent mosquitoes from breeding simply by decreasing the number of aquatic habitats or the volume of standing water [a2,1(−)]. Altering the distribution of aquatic habitats is also an important aspect of source reduction because extended prolonged oviposition cycles, caused by increasing the amount of time required for mosquitoes to locate oviposition sites, can reduce the basic reproductive rate of malaria [28]. Furthermore, source reduction can aggregate mosquito larvae and their predators, such as aquatic insects, into a smaller number of breeding sites and encourage aquatic insects to prey on mosquito larvae [a8,4(−)] [5]. Source reduction could indeed have played an important role in eradicating malaria from Israel, the USA and Italy [23,27] and in some local elimination programmes in Africa [26,29] by taking the following pathway [a2,1(−) x a8,2(+) x a9,8(+) x a11,9(+) = malaria incidence(−)]. Because the abundance and distribution of aquatic habitats are important determinants for local malaria transmission intensity and risk, source reduction can be more effective than applying larvicide [27].

While LLINs and IRS target mainly indoor-biting mosquitoes, source reduction can reduce both indoor- and outdoor-biting mosquitoes [24]. Because outdoor biting is becoming a more important feature of malaria transmission [30,31], the importance of source reduction is revisited, as this is one of the few effective strategies to reduce outdoor-biting mosquitoes [24]. However, decreasing availability of breeding sites can also reduce proliferation of predators of larvae [a2,1(−) x a4,2(+) x a8,4(−) = mosquitoes(+)]. Therefore, this negative impact on mosquito predators needs to be taken into consideration when forecasting the overall impact of source reduction on the increase in infective mosquitoes.

The impact of educational intervention in promoting source reduction and other interventions is compiled in the final subsection of Results.

### Insecticide and larvicide use

#### Insecticide use for adult mosquitoes (IRS)

Of the three major malaria control strategies (IRS, LLIN and prompt and effective treatment) promoted by the Roll Back Malaria Partnership (RBM) [32], two rely on chemicals (Figure 2). In order to directly suppress the abundance of adult mosquitoes by IRS, WHO approves the use of 12 insecticides of four chemical classes (organochlorines, organophosphates, carbamates, and pyrethroids) [33,34]. Several countries recently added IRS to their national malaria control plans in line with RBM [35], and 79 malaria-endemic countries reported the use of IRS for malaria prevention [1]. As a result, IRS has significantly reduced malaria vectors and malaria incidence [a9,3(−) x a11,9(+) = malaria incidence(−)] [36-40].

Two major challenges that chemical use has been facing in terms of reducing malaria incidence are disturbances to the natural balance, such as predator–prey relationships and mosquitoes’ insecticide resistance. Chemical insecticides and pesticides not only reduce the abundance of target organisms but also that of beneficial organisms, such as predators, either directly by toxicity or by eliminating their prey organisms [41,42]. Chemical use for malaria vector control can result in reduced abundance of predators, especially in aquatic larval habitats, and a subsequent increase in vector mosquitoes [a5,3(−) x a8,5(−) x a9,8(+) = infective mosquitoes(+)] [41,43,44].

The other major challenge is mosquitoes’ resistance to chemicals. Because chemical insecticide interventions have been scaled up during the past decade, mosquitoes’ resistance has been spreading worldwide [1,3,45]. In 2012, 64 countries reported resistance to at least one insecticide in one malaria vector in one study site, and most of the resistance was against pyrethroids [1]. Due to the heavy reliance on one class of insecticides, the pyrethroids, mosquitoes’ resistance to pyrethroids has been spreading at an exceptionally rapid rate, especially in Africa [1,46]. As a result, resistance began to threaten the sustainability of insecticide-based malaria control interventions [a7,3(+) x a3,7(−) x a9,3(−) x a11,9(+) = malaria incidence(+)] [3,4,47,48]. At the same time, there is a possibility that mosquito predators have developed or will develop insecticide resistance, which favours predator population and might suppress mosquito proliferation [a7,3(+) x a3,7(−) x a5,3(−) x a8,5(−) = mosquitoes(−)].

There is an urgent need to develop new vector control interventions. This is because the above-stated challenges exist and also because many of the anopheline species are not susceptible to current insecticide-based interventions such as IRS and LLINs, which target indoor-feeding/resting vectors [3,49]. New vector control interventions need to be developed and implemented, taking mosquito ecology into consideration, such as site preferences for feeding, resting (especially outdoor), and oviposition, mating behaviour, and sugar meal selection [3].

### Larvicide use

One of the most common interventions conducted to control mosquito larvae is the application of larvicide such as *Bacillus thuringiensis israelensis* (Bti) and *Bacillus sphaericus* (Bs) [24]. Several previous studies reported the effectiveness of larvicides in controlling malaria transmission. For example, hand-applied larviciding reduced transmission by 70-90% in Africa where the majority of aquatic mosquito larval habitats were defined and the aquatic surface was not too extensive [a8,3(−) x a9,8(+) x a11,9(+) = malaria incidence(−)] [24,50-52].

The application of larvicide for larval control can be effective in reducing not only the abundance of indoor-biting/resting mosquitoes, which IRS and LLINs target, but also of outdoor-biting/resting mosquitoes. However, several limitations have been reported. They include disturbance to local ecosystems such as predator–prey relationships [a4,3(−) x a8,4(−) = mosquitoes(+)] [41,53], mosquitoes’ resistance to larvicides [a7,3(+) x a3,7(−) x a8,3(−) = mosquitoes(+)] [54-56] and ineffectiveness in extensive water bodies [24,57]. In addition, Bti lacked inherent residual activity outside of potable container habitats, especially in habitats with turbid water or high organic loading [58].

### Biological control

Interest in formulating non-chemical approaches has been growing over the past four decades because of the limitations of chemical use, including mosquitoes’ insecticide resistance, disturbances to the ecosystem, and the health risks for human and domestic animals [5,59]. Current biological control tools that are considered most promising for malaria prevention include fungi, bacteria, larvivorous fish, parasites, viruses, and nematodes [60] (Figure 3). Among these, one of the most commonly used biological control agents is larvivorous fish, which are introduced to aquatic habitats of mosquitoes. Not only naturally occurring predators [61-64] but also introduced predators [24] can be effective in suppressing anopheline larval population.

Larvivorous fish, especially Gambusia (*Gambusia affinis*) and Guppy (*Poecilia reticulata*), are the most widely disseminated biological control agent in the world. Many of the introductions were made to control anopheline species that transmit malaria [65]. The usefulness of *G. affinis* in malaria control programmes was reported as early as the beginning of the 1900s in Europe, noting that the fish had a definite impact on the suppression of the disease [a8,12(−) x a9,8(+) x a11,9(+) = malaria incidence(−)]. Later in 1970, an extensive release programme was carried out in Iran, which demonstrated the important roles of *G. affinis* in malaria eradication [66]. The introduction of these larvivorous fish has been reported to be effective in controlling local malaria by recent studies as well, especially in Asia and Africa [67-69].

A major objection to the introduction of larvivorous fish has been their direct impact on native fish species through predation or their indirect impact through competition [a4,12(−)]. So far, more than 30 species of native fish and other aquatic invertebrates co-inhabiting the same waters have been adversely affected by the introduction of *G. affinis*[66]. Also, the introduction of *G. affinis* did not show good results in pits, riverbed pools, stone quarries, ponds, drains, rice fields, and irrigation drains alongside rice fields [70]. In addition, *G. affinis* have been reported to be little more effective or equal or less effective in mosquito control compared to native fish species they replace. In California, native *Cyprinodon macularius* had an equal effectiveness in mosquito control [71]. Application of other biological agents also involve limitations, which vary depending on the agent [60]. Although their effectiveness is promising, the use of these biological means needs to be planned carefully, taking their impact on the local ecosystem into consideration.

### Anti-malarials

Current malaria control measures directly targeting human beings rely heavily on a limited number of tools, particularly anti-malarial drugs (Figure 4) and LLINs, both of which have become less effective because of resistance. It was reported in 2011 that 79 countries/territories used artemisinin-based combination therapy (ACT) as first-line treatment for *Plasmodium falciparum* malaria [a11,13(−) = malaria incidence(−)]. For *Plasmodium vivax* malaria, it is recommended that chloroquine (combined with a 14-day course of primaquine) be used where it is effective, or an appropriate ACT in areas with chloroquine resistance [1].

Malaria treatment with ACT has been spreading over the world, but access to ACT at community level still needs to be improved. The number of ACT treatment courses delivered by manufacturers to the public and private sectors increased from 11 million in 2005 to 278 million in 2011. Surveys conducted in 12 African countries in 2010–2011 showed that about two thirds (median, 65%) of all febrile children treated with an anti-malarial received an ACT. A greater proportion of children received ACT at health facilities not in the community. Therefore, expanding appropriate malaria treatment to the community level is urgently needed [1].

In addition to insufficient access to appropriate treatment, resistance to artemisinin derivatives has been posing a serious threat to malaria treatment [a14,13(+) x a13,14(−) x a11,13(−) = malaria incidence(+)]. Parasites’ resistance to anti-malarial drugs arose from the extensive use and misuse of the drugs, particularly during the Global Malaria Eradication campaign, launched by WHO in 1955 [72]. So far, resistance of *P. falciparum* to artemisinin has been detected in Burma, Cambodia, China, Thailand, and Vietnam, [1,73-76]. Artemisinin resistance is a major threat to public health worldwide, especially to sub-Saharan Africa with the highest disease burden and inadequate systems for monitoring and containment of resistance [74].

One of the leading causes for the development of drug resistance is the spread of poor-quality anti-malarial drugs. It is very likely that widespread availability of counterfeit anti-malarials has been accelerating drug resistance in forested areas near the Thai-Cambodian border [77,78]. A recent review study found that up to 36% of anti-malarial drugs collected in Southeast Asia were falsified, and a third of anti-malarials collected in sub-Saharan Africa failed chemical assay analysis [79].

Intermittent preventive treatment (IPT) is also considered a cause of spreading resistance. IPT is a method proposed to reduce malaria morbidity and mortality by providing regularly spaced therapeutic doses of anti-malarials to individuals, regardless of their malaria infection status [80]. IPT has been shown to be effective in reducing clinical malaria cases in pregnant women, children and infants [81-84]. For example, it was shown that IPT targeting preschool children (age < six years) during the malaria transmission season markedly reduced clinical malaria cases, which occurred even in areas with high ITN use [81]. Despite its effectiveness, several studies suggested that IPT could accelerate the spread of resistance [85-87]. For example, IPT targeting infants is thought to accelerate the spread of resistant parasites in areas of low or unstable transmission and is more likely to accelerate the spread of resistance in high transmission areas than is IPT in adults [80].

To halt the spread of resistance, multiple strategies need to be employed. For example, it is crucial to improve facilities to check the quality of anti-malarial drugs and to strengthen drug-resistance surveillance and response systems. In western Cambodia (Pailin province), because resistance was found to both components of multiple ACT, special provisions for directly observed therapy using a non-artemisinin-based combination (atovaquone-proguanil) have been put in place [1]. Also, there is an urgent need for new anti-malarial drugs that can kill gametocytes, not the asexual blood stage of the parasite, to prevent malaria transmission [88]. In addition to improving diagnosis and access to inexpensive genuine medicines, raising consumer and health-worker awareness and knowledge about counterfeit drugs and the consequences of their use is urgently needed at the community level [74,79,89].

### Insecticide-treated bed nets/long-lasting insecticidal nets

ITNs, including LLINs, are considered to be the most prominent malaria preventive measure, especially in highly malaria-endemic areas [36,46,90,91] (Figure 5). To achieve Millennium Development Goal 6, which aims to reduce child mortality by 2015, ITNs are one of the most important measures to be taken [92]. ITNs provide personal protection as well as community protection by decreasing the biting rate and daily survival rate of malaria vector mosquitoes [a10,16(−) x a11,10(+) = malaria incidence(−)] [93-95] . ITNs can indirectly reduce malaria incidence through decreasing the infective mosquito population by affecting their survival [a10,16(−) x a9,10(+) x a11,9(+) = malaria incidence(−)]. Reduced biting rate and daily survival rate can also affect the uptake of *Plasmodium* to mosquitoes and prevent mosquitoes from becoming infective [a10,16(−) x a6,10(+) x a9,6(+) = infective mosquitoes(−)] [96]. ITNs have been shown to be effective in reducing mortality from malaria in previous studies and randomized controlled trials [90,97-99]. A previous systematic review reported that ITNs significantly reduce the incidence of malaria compared to no nets and untreated nets in areas with stable malaria as well as with unstable malaria [a10,16(−) x a11,10(+) = malaria incidence(−)] [90].

During the past decade, ITN coverage has increased substantially. By 2012, 117 countries, including 34 in Africa, had adopted the WHO recommendation to provide ITNs to all persons at risk of malaria. A total of 88 countries, including 39 in Africa, distribute ITNs free of charge [100]. Distribution of ITNs has increased exponentially from 2007, especially in sub-Saharan Africa with household ownership of at least one ITN becoming an estimated 54% by 2013 [45,100].

However, the number of ITNs delivered in 2011 and 2012 was below the number of ITNs required to protect all populations at risk [1]. Although rapid increase in ITN coverage has occurred in some of the poorest countries in Africa, coverage remains low among populations at risk. Among 44 African countries, only four have achieved ITN ownership coverage of 80% or greater. Countries with large populations at risk of malaria, such as Nigeria, continue to have low coverage. Overall, ITN ownership coverage was 32.8%, and ITN use in children under five was 26.6% among 44 African countries in 2008 [98]. In addition, the proportion of the population sleeping under an ITN has been reported to be higher in wealthier, urban areas and lower among older children [1,101,102]. ITNs provided through free mass campaigns can actually preferentially cover children from the poorest quintile homesteads [102], and disparities in ITN access are expected to diminish as programmes move towards universal coverage [1].

Inappropriate use of ITNs is another serious issue related to malaria prevention. Household surveys conducted in Africa from 2003–2011 indicated that approximately 90% of the population with access to an ITN within the household actually used it. However, the population that used available nets included households in which nets were used beyond their assumed capacity as well as those in which nets were not used to full capacity [1]. ITN misuse has been increasingly reported. For example, ITNs are used as sleeping mats, for fishing or for drying fish, to protect crops and plants, as wedding veils, and as chicken coops [103-105]. Although a question remains as to whether the ITN misuse impedes ongoing malaria control efforts [103,104], further work is needed to ensure that all available nets are fully and properly utilized [1].

Resistance to pyrethroids, used for treating bed nets, is threatening the effectiveness of ITN use in reducing malaria incidence [a7,16(+) x a16,7(−) x a10,16(−) x a11,10(+) = malaria incidence (+)], [a7,16(+) x a16,7(−) x a10,16(−) x a9,10 (+) x a11,9(+) = malaria incidence(+)], and [a7,16(+) x a16,7(−) x a10,16(−) x a6,10 (+) x a9,6(+) x a11,9(+) = malaria incidence(+)] [46,48,91,106]. For the treatment of bed nets, only six insecticides, all of which belong to the pyrethroid class, are allowed by WHO (WHO Pesticide Evaluation Scheme). The use of pyrethroids in malaria vector control has increased dramatically in the past decade through the scale up of ITN distribution programmes and IRS campaigns in Africa [46]. In addition, pyrethroids are widely used to control agricultural pests, which can pose additional selection pressure on mosquitoes when insecticides contaminate larval habitats. This intensive exposure to insecticides has inevitably resulted in the evolution of insecticide resistance in anopheline mosquitoes [34], and the resistance alleles have been spreading at an exceptionally rapid rate throughout Africa [46].

In spite of the rapid spreading of pyrethroid resistance, few studies have examined the impact of ITN use on malaria control. Controversy remains about the epidemiological significance of current levels of resistance in sub-Saharan Africa [46]. A recent study conducted in Benin demonstrated that resistance seriously threatens ITN-based malaria control interventions because ITNs provide little or no protection once vectors became resistant and netting acquires holes [106]. However, another study in seven locations in Africa reported that ITNs were cost effective for malaria control even in areas with strong pyrethroid resistance [91].

### Non-chemical personal protection measures

Personal protection measures against mosquito-borne diseases with non-chemical approaches are considered to be potentially important [107] (Figure 6). Such measures include wearing light-coloured clothing, long trousers, long-sleeved shirts, and avoiding areas with high mosquito density. The effectiveness of such measures in reducing malaria incidence has not been well examined and needs to be studied. However, the improvement of house design has already been proven to be effective as a personal protection measure that does not rely on chemicals.

Improving the domestic environment, such as house design and screening, can be a non-chemical, complementary approach to increasing personal protection against indoor-biting malaria vectors and interrupting the malaria transmission cycle [a10,17(−) x a11,10(+) = malaria incidence(−)] [108-111]. Transmission of malaria and other mosquito-borne diseases can be facilitated by poor house design that favours mosquito entry [112-114]. For example, the lack of window/door screening, presence of eave gaps and lack of a ceiling have been reported to enhance mosquitoes’ entry into houses [109,115-118].

It has been demonstrated that the improvement of house design significantly contributes to the reduction in mosquito density inside houses and to the control and reduction of malaria vectors [a10,17(−) x a9,10(+) = infective mosquitoes(−)]. The method includes screening (even with used bed nets or untreated shade cloth for agricultural use) [109,119-122], blocking all potential house entry points for mosquitoes [116,117] and building houses on stilts [123].

### Educational intervention (awareness)

Community participation is vital for successful malaria control [2,124-127]. A recent review study identified the three most influential factors for community participation: knowledge and perception of disease, multisectoral collaboration and integration of programme(s) into broader development goals, and decentralization of power and resources and the use of community assets [2]. In order to raise community awareness and to involve the community in malaria control interventions, a variety of educational programmes have been conducted in malaria-prone countries (Figure 7).

The effectiveness of such community-based educational programmes in promoting malaria control with community participation has been reported by several studies. For example, a study from Nigeria demonstrated that it was health education, not free distribution, that significantly increased the use of ITNs among community residents [a16,15(+)] [128]. In Ethiopia, a cluster randomized trial demonstrated that the burden of malaria among children under five (examined by mass blood investigation) was significantly reduced by training household heads on the utilization of LLINs [a16,15(+) x a10,16 (−) x a11,10(+) = malaria incidence(−)] [129].

Educational interventions targeting community residents and community health workers have improved community actions to promote early diagnosis and treatment of malaria [a13,15(+)]. Recent studies from Nigeria indicated that a reduction in the incidence of malaria can be achieved by conducting training programmes for caregivers of children under five, which improved their knowledge, home management of malaria and referral practices for severe malaria [a13,15(+) x a11,13(−) x = malaria incidence(−)] [130,131]. Another study reported that a treatment guideline for the effective case management of malaria for children at home, developed by the joint efforts of researchers and community members, not only built capacity at the community level but also increased the acceptability and ownership of such materials [132]. In addition, training of community health workers has been reported to be effective in improving malaria diagnosis and treatment at the community level [a13,15(+)]. In Cambodia, community residents were trained as Village Malaria Workers and became effective in diagnosing malaria with rapid diagnostic tests (RDTs) and prescribing anti-malarials to malaria patients [133]. In Uganda, lay community health workers were trained and successfully diagnosed and treated malaria and pneumonia in children [134]. Training drug vendors, who can play a role in spreading information within a community, was also shown to be effective in improving prompt and appropriate treatment of malaria and referral of severe cases [135].

Source reduction was also promoted by community residents who were motivated and trained by different educational interventions [a1,15]. A community-based educational intervention, which was conducted for rice farmers in Sri Lanka, kept high participation rates and had a significant positive impact on the knowledge and varieties of actions farmers took for mosquito control and mosquito-borne disease prevention [136]. The farmers’ environmental management activities, including source reduction, were demonstrated to be effective in reducing the density of adult female anophelines [a1,15(+) x a2,1(−) x a8,2(+) = mosquitoes(−)] [5].

Effective implementation of IRS and application of larvicide also need educational programmes to improve community understanding and acceptance by community residents [a3,15(+)]. Previous studies reported that community residents’ understanding of the function of the IRS, especially its effectiveness and unwanted side-effects, was related to their compliance with the IRS programme [137-139]. One of the studies reported that the most frequent suggestion for improving community acceptance of IRS was to increase the understanding of the objectives of spraying in the communities [138].

Biological control of vectors and personal protection from malaria depends on community understanding, which educational intervention can improve. A previous study demonstrated that the successful implementation of biological control using fish, *Toxorhynchites* mosquitoes, *Notonecta* species, predatory copepods, entomopathogenic bacteria, and the fungus *Lagenidium giganteum* depended on the community’s in-depth understanding of the ecology of these agents and targeted species [140]. Another study showed that an educational intervention increased community residents’ activities to implement biological control using oil, salt and fish [a12,15(+)] [136]. Other previous studies have demonstrated the effectiveness of educational intervention in improving non-chemical personal protection measures taken by community residents and community health workers, such as wearing long-sleeved shirts and long trousers and adding windows or door nets to houses [a17,15(+)] [136,141]. Several historical reports also describe that education was an essential part of mosquito control activities throughout the United States, especially in early 20^th^ century [142].

School education can also be an important strategy for community-based malaria control. A previous study in Ghana demonstrated that school-based participatory health education decreased malaria prevalence among school children and improved knowledge and practices of adults in the community [143]. However, a recent cross-country study reported that school textbooks of primary and lower secondary schools rarely covered knowledge and skills for malaria prevention and treatment [144]. Utilization of school education could further raise community awareness and encourage school children, their parents, and other community members to take additional actions for malaria control.

## Discussion

This is the first study that has demonstrated how the impact of community-based malaria control interventions on malaria incidence can be examined by qualitative analysis, specifically loop analysis, combined with an extensive review of the literature that analyses each pathway. The sign digraphs developed in this study give a more complete picture of the complex system that can be created by a variety of malaria control efforts at the community level. By carefully mapping out relevant variables in the system, the digraph explains interactions and correlations among the variables involved. The sign digraphs show a variety of possible pathways through which each intervention can influence local malaria incidence. Combining sign digraphs of plural interventions can demonstrate changes in the variety and number of pathways as well as interactions between interventions.

Sign digraphs can demonstrate where uncertainties might exist in the complex system and what kinds of research are necessary to better understand how community-based malaria control interventions might influence local malaria incidence. For example, the sign digraph of source reduction (Figure 1), shows a negative impact of the reduction in mosquito breeding sites on mosquitoes’ predators, which might eventually increase mosquito larval density in the long run [a2,1(−) x a4,2(+) x a8,4(−) = mosquitoes(+)]. However, most previous studies focused only on source reduction’s direct, short-term impact on mosquito larvae, and few studies have examined its impact on the ecology and density of predators. The diagram suggests that both pathways ([a2,1(−) x a8,2(+) = mosquitoes(−)] and [a2,1(−) x a4,2(+) x a8,4(−) = mosquitoes(+)]) need to be studied in order to accurately evaluate the overall impact of source reduction on larval mosquitoes.

A sign digraph can also be useful to qualitatively examine the mechanisms of how combined effects of plural interventions can be generated. Recent studies have demonstrated that the combination of IRS and ITN use resulted in greater reductions in malaria incidence compared to the use of IRS or ITNs alone [36,145,146]. However, the sign digraph (Figure 2) shows that insecticide use is interlinked with ITN use through insecticide resistance and that the combined use of these measures can each diminish the effectiveness of the other by further accelerating the spread of resistance. Although some studies have suggested that the insecticides used for IRS and ITNs in the same region should belong to different classes to prevent the development and spread of insecticide resistance [36,146], few studies have monitored how insecticide resistance progresses with the combination of the two interventions compared to the use of one of them. This way, a sign digraph demonstrates benefits and drawbacks of combining plural interventions as well as the need of further research on issues that have not been well examined.

Several vital issues for the effective implementation of each intervention, including sustainability and cost-benefit analysis, need to be considered when interpreting a sign digraph. For example, although a sign digraph explains pathways through which community awareness and educational interventions can influence local malaria incidence (Figure 7), it does not show how long the impact of the educational intervention or raised awareness could last. The literature search revealed few studies that evaluated the long-term impact of educational interventions on community awareness, actions and malaria incidence. Such research is urgently needed. A deeper understanding of the sustainability of community participation is vital to encourage communities to continue their malaria control activities even when malaria incidence decreases with remaining transmission occurring in defined foci [147,148].

The costs and benefits of each intervention also need to be taken into consideration. In most cases, community awareness and cost-benefit analysis conducted by community members can vary over time. Whether or not a community-based malaria control intervention can be sustained depends on a variety of issues, especially the cost of different materials and activities necessary to conduct the intervention. Such cost can vary by season because it not only includes direct costs to purchase materials or tools but also community members’ time taken from other activities such as agriculture and fishing. As local malaria incidence decreases, it becomes increasingly difficult to sustain community awareness and participation [2,11,12] and to persuade governments to allocate funding to maintain effective interventions. Since 1930, 75 resurgences of malaria have been recorded, nearly all of which were linked to the scaling back of interventions [76,149]. Considering such variations over time when interpreting a sign digraph could strengthen understanding of the mechanisms of how each intervention might work to decrease local malaria incidence.

One of the limitations of the study is that, mainly due to the nature of qualitative analysis, magnitudes of the interactions between variables were not examined. Therefore, it was impossible to quantitatively compare the impact of different interventions on malaria incidence and to examine the combined effects of plural interventions on malaria incidence. Also, to examine the interaction between variables involved in the system, different kinds of literature had to be combined regardless of their methods of analysis. Some studies conducted quantitative analysis while others only provided descriptive information without statistics showing the extent of changes. Publication bias might have influenced this study’s analysis to some extent because most of the articles have dealt with the positive or negative impact of one variable on another rather than null effects.

Despite these limitations, this is the first study to qualitatively review the impact of malaria control interventions on malaria incidence, using the loop analysis method. Without relying on numerical data, this study was able to describe pathways through which each intervention could influence malaria incidence on the basis of the qualitative patterns of the interactions among variables in complex systems. It contributed to a better understanding of the mechanisms of how each malaria control intervention could influence malaria incidence, examined the associations and correlations among a variety of factors involved, and explained how unexpected consequences may have occurred. This methodology can be utilized not only by researchers but also by community leaders, local health programme and project officers, and policy makers to better plan and conduct their community-based malaria control interventions. It is also applicable to future studies to review the impact of malaria and other disease control interventions at community, national, and global levels.

## Competing interests

The authors declare that they have no competing interests.

## Authors’ contributions

JY conceived the study, conducted analysis and literature review, and wrote the manuscript. MJ contributed to the interpretation of the analysis and improved the manuscript. RL provided guidance to conduct analysis and improved the manuscript. All authors read and approved the final draft.
